# Rapid Amygdala Kindling Causes Motor Seizure and Comorbidity of Anxiety- and Depression-Like Behaviors in Rats

**DOI:** 10.3389/fnbeh.2016.00129

**Published:** 2016-06-22

**Authors:** Shang-Der Chen, Yu-Lin Wang, Sheng-Fu Liang, Fu-Zen Shaw

**Affiliations:** ^1^Department of Neurology, Kaohsiung Chang Gung Memorial Hospital, Chang Gung University College of MedicineKaohsiung, Taiwan; ^2^Center for Translational Research in Biomedical Science, Kaohsiung Chang Gung Memorial Hospital, Chang Gung University College of MedicineKaohsiung, Taiwan; ^3^Department of Computer Science and Information Engineering, National Cheng Kung UniversityTainan, Taiwan; ^4^Institute of Medical Informatics, National Cheng Kung UniversityTainan, Taiwan; ^5^Department of Psychology, National Cheng Kung UniversityTainan, Taiwan

**Keywords:** amygdala kindling, afterdischarge, anxiety, depression, anhedonia

## Abstract

Amygdala kindling is a model of temporal lobe epilepsy (TLE) with convulsion. The rapid amygdala kindling has an advantage on quick development of motor seizures and for antiepileptic drugs screening. The rapid amygdala kindling causes epileptogenesis accompanied by an anxiolytic response in early isolation of rat pups or depressive behavior in immature rats. However, the effect of rapid amygdala kindling on comorbidity of anxiety- and depression-like behaviors is unexplored in adult rats with normal breeding. In the present study, 40 amygdala stimulations given within 2 days were applied in adult Wistar rats. Afterdischarge (AD) and seizure stage were recorded throughout the amygdala kindling. Anxiety-like behaviors were evaluated by the elevated plus maze (EPM) test and open field (OF) test, whereas depression-like behaviors were assessed by the forced swim (FS) and sucrose consumption (SC) tests. A tonic-clonic convulsion was provoked in the kindle group. Rapid amygdala kindling resulted in a significantly lower frequency entering an open area of either open arms of the EPM or the central zone of an OF, lower sucrose intake, and longer immobility of the FS test in the kindle group. Our results suggest that rapid amygdala kindling elicited severe motor seizures comorbid with anxiety- and depression-like behaviors.

## Introduction

Temporal lobe epilepsy (TLE) is the most drug-resistant type of adult focal epilepsy. A considerable portion of TLE patients (20–50%) have comorbidity of anxiety and/or depression (Kanner, 2007; Desai et al., 2010). The epileptic focus in TLE patients or TLE-like animal models often resides in a mesial temporal structure, such as amygdala, hippocampus, or both. Previous studies have indicated that the amygdala is a critical component of the abnormal circuitry underlying temporal lobe seizures (Goddard, 1983; Pitkänen et al., 1998). The amygdala is among the more susceptible regions to kindling (Kalynchuk, 2000; Morimoto et al., 2004). Thus, amygdala kindling is a popular experimental model for TLE with partial-onset and secondarily generalized convulsion (Goddard, 1983).

Conventional amygdala kindling (Goddard, 1983; Lothman and Williamson, 1994), where two electrical stimulations are delivered per day, has been investigated on the process of epileptogenesis and changes in interictal emotionality (Kalynchuk, 2000). The amygdala is a part of the limbic system and is associated with the modulation of emotion and fear. Kindling of the right-side amygdala of animals elicits anxiety-like behavior (Adamec and Morgan, 1994; Helfer et al., 1996; Hannesson et al., 2008). Conventional amygdala kindling did not aggravate depression-like behaviors, such as anhedonia or despair mood (Helfer et al., 1996; Wintink et al., 2003; Adamec et al., 2004). Lacking comorbidity of depression-like behavior in the conventional kindling model strikingly differs from observations in TLE patients (Kanner, 2007) or other TLE-like animal models, e.g., kainate-induced model (Tchekalarova et al., 2013) or pilocarpine-induced model (Mazarati et al., 2008).

Rapid amygdala kindling, where up to 24 stimulations are given each day (Lothman et al., 1985; Lothman and Williamson, 1994), is a variation of the conventional kindling model with the advantage that kindling can occur over few days and shows neuronal plasticity in the limbic region (Ebert and Löscher, 1995; Smith et al., 2005). The rapid kindling process has been used to assess anticonvulsants or alternative therapeutics (De Smedt et al., 2007; Shahpari et al., 2012; Shojaei et al., 2014). Spike patterns of epileptiform discharges with regard to the severity of motor seizures have a similar development between conventional and rapid amygdala kindling (Wang et al., 2016). However, rapid kindling of the amygdala causes epileptogenesis accompanied by anxiolytic response in early isolation of rat pups (Jones et al., 2009) or depressive behavior in immature rats (Mazarati et al., 2007). Phenomena of psychiatric comorbidity in the rapid kindling model seem to differ from those of conventional kindling model in adult rats (Adamec and Morgan, 1994; Helfer et al., 1996; Wintink et al., 2003; Adamec et al., 2004; Hannesson et al., 2008) or patients with TLE (Kanner, 2007; Desai et al., 2010). These controversial results may arise from using a specific group (immature or early separated stress) in the rapid kindling model. To our knowledge, emotional dysfunctions of the rapid kindling model in adult rats are still unknown.

In the present study, the rapid amygdala kindling was performed in adult rats. We hypothesized that rapid amygdala kindling caused epileptiform activity accompanied by anxiety- and depression-like behaviors as similar to most TLE patients with psychiatric comorbidity. The results may advance our understanding in progression of epileptiform activity and emotional disturbance through rapid amygdala kindling.

## Materials and Methods

Adult male Wistar rats (8–15 weeks, 350–500 g) were used. Animals were maintained in a sound-attenuated room under a 12-h light/dark cycle (lights on at 06:00–18:00) with food and water available *ad libitum*. Rats were randomly assigned to the kindle and control groups. The experimental procedures were reviewed and approved by the Institutional Animal Care and Use Committee. All experiments complied with guidelines recommended by NIH (USA) on the ethical use of animals.

### Surgery

Rats were anesthetized by sodium pentobarbital (50 mg/kg, i.p.). Subsequently, the dorsal surface of the head was shaved, and the rat was placed in a standard stereotaxic apparatus. A midline incision was made for placing stainless steel screws and Teflon-insulated 0.2-mm diameter stainless steel microwires (#791600, A-M Systems, Sequim, WA, USA). Animals were equipped with stainless steel screws connected by insulated wires to a microconnector for electrocorticographic (ECoG) recordings. Screw electrodes were bilaterally implanted over the area of the frontal cortex (2 mm anterior, 2.5–3.5 mm lateral from the bregma). Three stainless steel microwires, two for stimulation and one for recording, were implanted into the right basolateral amygdala (2.6 mm posterior and 4.8 mm lateral from the bregma, 8.5 mm ventral from the surface). The stimulated microwires were made of 0.5 mm tip exposure and the tip separation of 0.5 mm (Helfer et al., 1996). Two twisted microwires for recording were implanted into the left basolateral amygdala. A ground electrode was implanted 2 mm caudal to lambda. Screw electrode and microwire assemblies were attached to the skull with dental acrylic. Animals were given antibiotic (chlortetracycline) after suturing and housed individually in cages for a recovery period of >1 week.

### Kindling

Stimulation of the right-side amygdala was applied by a constant current stimulator (Grass 48, Grass Technique, West Warwick, RI, USA). Activities of bilateral frontal cortices and amygdala were recorded during the kindling. On the pre-kindling period, a 2-s, 80-Hz monophasic square-wave stimulus of 1 ms per pulse was used to determine the afterdischarge (AD) threshold. The stimulus intensity began at 50 μA, and was subsequently increased by 25 μA steps every 30 min until at least 5-s AD was elicited. Therefore, the intensity that ≥2 of three stimulations produced ADs was defined as the AD threshold.

One day after determination of the AD threshold, amygdala kindling was carried out. Stimulations of 200 μA, 80 Hz, 1 ms pulse width for a total duration of 2 s were delivered from an isolated constant current stimulator. Two consecutive kindling stimulations were separated by 30 min, and a maximum of 20 stimulations were given daily (Racine et al., 1973; Wang et al., 2016). The intensity of the stimulated current for kindling was set at 200 μA to control the degree of current spread into adjacent brain regions (Ehlers and Koob, 1985). The behavioral stages observed after each stimulus were classified using Racine’s standard 5-stage scale (Racine, 1972): stage 1, facial movements; stage 2, rhythmic head movements, head nodding; stage 3, unilateral forelimb clonus; stage 4, bilateral forelimb clonus and rearing; stage 5, falling and tonic-clonic convulsion. AD duration was the total time of epileptiform activities at the stimulated amygdala electrode, including the stimulation period.

### Recording of Brain Activities

Bilateral frontal ECoGs and field potentials of bilateral basolateral nuclei of the amygdala were amplified and filtered (0.7–300 Hz) through a multichannel amplifier (Shaw et al., 2002). A grounded metal plate was placed under the recording chamber to reduce electromagnetic interference (Shaw et al., 2003). The amplified signals were digitized at 1000 samples/s (USB6009, National Instruments, TX, USA) and stored on a hard disk for offline analysis. The entire software for data acquisition and analysis was under LabVIEW platform (National Instruments, TX, USA).

### Experimental Procedure

**Figure 1:**
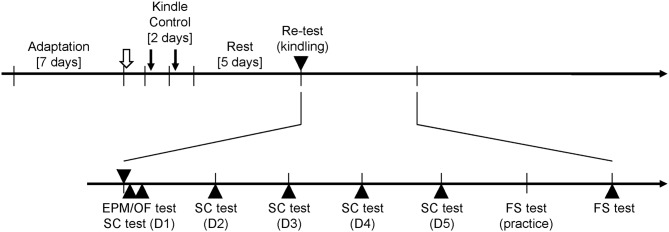
**Experimental paradigm for the rapid amygdala kindling and behavioral tests.** The afterdischarge (AD) threshold of amygdala kindlings (open arrow) is measured after 1 week adaptation for the recording apparatus. During the rapid kindling process, 20 amygdala stimulations (downward arrows) are performed in the kindle group. The control group has same setups as the kindle group but receives no stimulation. Five days later, the kindle group receives a kindling retest (▾), and the control group has similar setting but no stimulation. All rats perform the elevated plus maze (EPM) test or open field (OF) test 60 min after the retest stimulation. Thirty minutes later, the sucrose consumption (SC) test are carried out for 5 days. One day later, the forced swimming (FS) test is practiced for 10 min. Next day, a 5-min testing is performed.

Two weeks after surgery, all rats were placed in a recording room. To allow rats to habituate the experimental setting, each rat was placed in the recording apparatus 30 min/day for 7 days. The experiments of determining AD thresholds and all behavioral tests were performed at 13:00–17:00 to minimize circadian influences. AD thresholds of all rats were determined first. Thereafter, 20 amygdala stimuli were repeatedly done in the kindle group for 2 days. In the control group, all rats had the same setup as the kindle group did except for the amygdala stimulation. Five days later, a 200-μA stimulation was employed to ascertain Racine’s stage in the kindle group, whereas the control group was set with a connector assembly for 10 min without a re-test stimulation. The rat was excluded if Racine’s score of the re-testing stimulation was not same as the Racine’s score of the 40th stimulation. Sixty minutes later, the elevated plus maze (EPM) test was performed followed by the sucrose consumption (SC) test with interval of 30 min. One day later, the SC test was performed daily for 4 days. Subsequently, a 15-min habituation was done first, and a 5-min forced swim (FS) test next day. A schematic flowchart of the entire experimental procedure is shown in Figure 1.

In addition, we carried out the second experiment for the open field (OF) test. The procedure of the second experiment was similar to the first experiment. The kindle group performed the OF test 60 min after the re-testing kindling stimulation.

### Behavioral Test

Three behavioral tests were used to evaluate the anxiety- and depression-like behaviors of rats (Jones et al., 2008; Huang et al., 2012). The EPM test and OF test are used to measure anxious status. The FS test is used to assess the duration of immobility, which is the experimental analog of a despair-like status. The SC test is a measure of the “hedonic” state of an animal, or the ability to experience pleasure. A decreased sensitivity to reward (or anhedonia) is a fundamental feature of clinical depression.

#### Sucrose Consumption Test

In the SC test, each rat was placed in a test cage identical to its home cage. Consumption of a 20% sucrose solution was recorded for 15 min. Sucrose intake was measured by reweighing a preweighed bottle at the end of the test. Prior to testing, rats were not deprived of food and water. In this study, fluid intake over 5 days was used for statistical evaluation of differences in the two groups of rats. Decreased sucrose intake, i.e., anhedonia, is a validated index of a depression-like state (Jones et al., 2008).

#### Forced Swim Test

Apparatus of the FS test was a plastic cylinder 47 cm in height with a 38-cm inside diameter containing 38 cm of water at 25 ± 1°C. The FS test consisted of two phases. Rats were individually forced to swim in a plastic cylinder for 15 min on the first day. The 5-min test sessions began 24 h later. The duration of immobility, including passive swimming, was measured. The criterion for passive swimming was floating vertically in the water while making only those movements necessary to keep the head above the water. The active swimming represented more vigorous activity than swimming: strong movement of all four limbs, jumping, etc. Increased immobility in the forced swimming (FS) test is indicative of depression-like behavior (Cryan et al., 2005).

#### Elevated Plus Maze Test

The EPM consisted of black polypropylene plastic which was elevated 68 cm above the floor. Each maze arm extended 45 cm from the junction area, which measured 13 × 13 cm. The open and closed arms (CAs) were 13 cm wide, and the closed maze arms had walls extending 25 cm from the junction area. During testing, each rat was placed in the central square facing an open arm (OA) and was allowed 5 min to freely explore the maze. The alleys of the maze were thoroughly cleaned with an ethanol solution (60% volume) after the removal of each rat. Time spent in OAs and the numbers of open and CA entries were measured. The percentage of entries in OAs was calculated according to the following formula: percentage of OA entry = (number of entries into the OAs/number of open + CA entries) × 100. Either the percentage of venturing into OAs and/or the amount of time spent in OAs are validated for anxiety (Rodgers and Dalvi, 1997).

#### Open Field Test

The test cage was comprised of black acrylic plastic that formed a square 99 × 99 cm with a wall height of 45 cm. The box was divided into nine equal squares measuring 33 × 33 cm. Recording was done in a room illuminated by a ceiling red fluorescent light (40 W). Rats were placed in the recording room 30 min before testing. Each of the rats was placed in the center zone of an OF at the beginning and allowed it to explore the maze for 5 min. The following behavioral elements were quantified: frequency of the central zone (CZ) entered and total movement in the recording cage. Anxiety-like behavior in the OF test is triggered by the agoraphobia (while rats were forced into the arena which is very large relative to the rats’ nature). The low number of crossing the CZ demonstrates animal’s anxiety (Prut and Belzung, 2003).

### Histological Identification

Each animal was deeply anesthetized with sodium pentobarbital (60 mg/kg, i.p.). A transcardiac perfusion was performed with 0.9% saline followed by 3.5% paraformaldehyde in 0.1 M phosphate buffer at pH 7.4. The brain was then removed and post-fixed in the same fixative with 20% sucrose overnight at 4°C. Brain sections in the coronal plane were cut at 40 μm thicknesses. The positions of electrode tips were plotted on sections of the rat atlas of Paxinos and Watson.

### Statistical Analysis

The AD thresholds of the two group were assessed by the independent *t* test. The correlation coefficient was used to evaluate the relationship between the AD threshold and the stimulated number attaining Racine’s stage 5. Racine’s score was assessed by 1-way repeated measures analysis of variance (ANOVA) on ranks. The AD durations and indexes of the SC test were evaluated by 2-way repeated measures ANOVA with one factor repetition, if appropriate, followed by *post hoc* *t* test with Bonferroni correction. Indexes of the EPM test, OF test, and FS test were assessed by the independent *t* tests.

Another interesting information was how many animals were comorbid with severe anxio-depressive behaviors. The present study calculated the rat number of the kindle group that the behavioral indexes exceeded the mean ± 99% confidence interval of behavioral indexes of the control group. Data was expressed as the mean ± standard error of the mean (SEM). A two-tailed significance level was set at *p* < 0.05.

## Results

**Figure 2:**
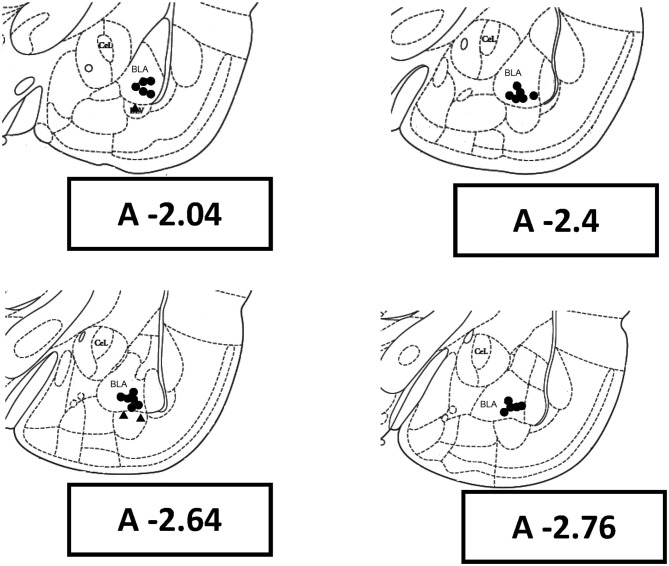
**Rat brain coronal sections with reference to the bregma show histological reconstruction of the stimulation sites in kindle animals.** BLA, basolateral amygdaloid nucleus, anterior; CeL, centrolateral nucleus.

In the present study, 58 rats were used. Five rats did not complete the experiment due to de-attachment of dental cement from the head under amygdala kindling. ADs were unable to be elicited from three rats through amygdala kindling, which was due to electrode placement outside the basolateral nucleus of the amygdala. The histological data showed that electrode tips of 25 rats were located in the basolateral nuclei of the right amygdala (Figure 2). Finally, 50 rats (control = 25, kindle = 25) were used in the first experiment for further analysis. In the second experiment, 30 rats (control = 15, kindle = 15) were used to explore anxious state in terms of the OF test.

### Kindling

The AD thresholds of the kindle and control groups were 153.0 ± 27.3 and 153.0 ± 26.3 μA, respectively. AD thresholds of the two groups had no significant difference. The AD thresholds were in the range of 100–200 μA, which was lower or equal to the current intensity of the rapid amygdala kindling.

**Figure 3:**
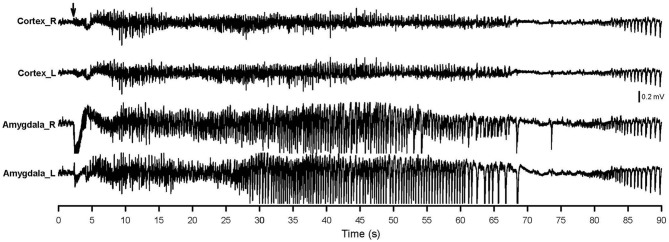
**AD of the 40th amygdala stimulation.** The right-side amygdala stimulation is indicated by an arrow. Cortex_R, right-side cortex; Cortex_L, left-side cortex; Amygdala_R, right-side amygdala; Amygdala_L, left-side amygdala.

Figure 3 shows the AD of the 40th stimulation in a rat. An extremely dynamic change in pattern and amplitude of waveforms was observed. Aberrant brain activity of >400 μV was observed, accompanied by a tonic-clonic convulsion 30–50 s after stimulation. Brain activity became a brief silence immediately after the AD then followed by postictal epileptiform discharge. The amygdala showed a strongly aberrant discharge compared to the cortex.

**Figure 4:**
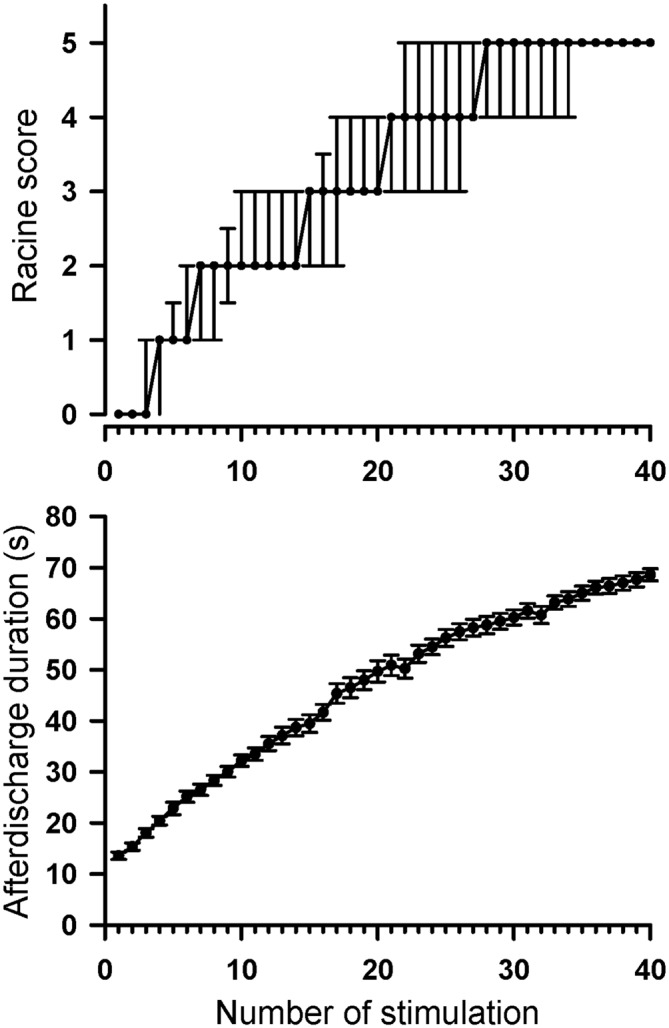
**Progression of Racine’s seizure score and AD duration throughout 40 amygdala stimulations in the control and kindle groups.** The data are presented with the median ± 25–75 percentile on Racine’s score and the Mean ± SEM on AD duration.

Throughout the rapid kindling process, rats progressively developed a tonic-clonic stage five convulsion as the stimulation number increased (Figure 4). All rats in the kindle group showed Racine’s stage 5 (tonic-clonic convulsion) after 40 right-side amygdala stimulations. The Racine’s score significantly increased as the stimulation number increased (*χ*^2^ = 903, *p* < 0.001). In addition, the AD duration became significantly longer as the amygdala kindling number increased (*F*_(39,936)_ = 334.7, *p* < 0.001).

Intuitively, rats with a low AD threshold might receive over-stimulation for a constant 200-μA kindling, which might result in attaining Racine’s stage 5 quickly. There was however no significant correlation between the AD threshold and the stimulating number attaining Racine’s stage 5 (*r* = −0.24, *p* = 0.27).

### Sucrose Consumption Test

**Figure 5:**
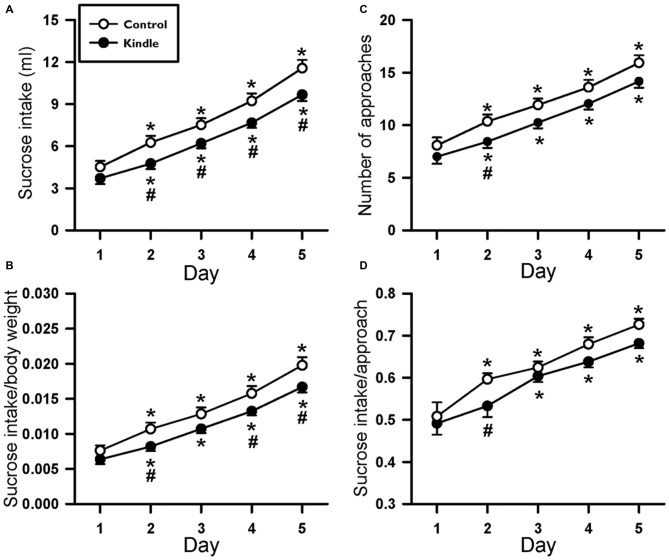
**Changes of the sucrose intake (A), sucrose intake normalized by body weight (B), number approaching to the bottle (C), and sucrose intake per approach (D) of the 5-day SC test in the control and kindle groups.** The data are presented with the Mean ± SEM. *p < 0.05 vs. control, ^#^*p* < 0.05 vs. first day.

Fluid intake during the SC test progressively increased as time passed in the two groups (Figure 5). Obviously, the kindle group had a significantly lower sucrose intake than that of the control group. Sucrose intake had significant differences in the factors of time (*F*_(4,249)_ = 228.0, *p* < 0.001) and treatment (*F*_(1,249)_ = 6.173, *p* = 0.017). There was no difference in SC between the two groups on the first day, but the kindle group had a significantly lower sucrose intake than the control group for the following 4 days (Figure 5A). More than 40% of the kindle group showed a remarkable lower sucrose intake than the mean −99% confidence interval of the control group in the last 4 days of the SC test (Table 1). The sucrose intake normalized by body weight showed significant differences in the factors of time (*F*_(4,249)_ = 199.2, *p* < 0.001) and treatment (*F*_(1,249)_ = 4.885, *p* = 0.032). The kindle group had a lower sucrose intake than the control group. In particular, there was no difference in normalized SC on the first day, but the kindle group showed significantly lower normalized sucrose intake than the control group at the 2nd, 4th, and 5th days (Figure 5B).

**Table 1:** **Rat number in the kindle group beyond the range (Mean ± 99% confidence interval) of the four behavioral tests of the control group**.

Behavior	Number (ratio)
Elevated plus maze test (*n* = 25)	
OA-duration	10 (40%)
OA/CA entry ratio	12 (48%)
Open field test (*n* = 15)	
Number crossing CZ	6 (40%)
Forced swim test (*n* = 25)	
Immobility	20 (80%)
Sucrose consumption test (*n* = 25)	
Sucrose intake (day1)	10 (40%)
Sucrose intake (day2)	11 (44%)
Sucrose intake (day3)	12 (48%)
Sucrose intake (day4)	11 (44%)
Sucrose intake (day5)	12 (48%)

*CA, closed arm; CZ, central zone; OA, open arm*.

We also analyzed the approaching number during the SC test. The approaching number showed a significant difference in the factor of time (*F*_(4,249)_ = 167.4, *p* < 0.001). The treatment factor almost attained a significant level (*F*_(1,249)_ = 3.835, *p* = 0.056). The kindle group had a remarkable lower frequency of approaching the sucrose bottle compared to the control group. In addition, the sucrose intake per approach made a significant difference in the factor of time (*F*_(4,249)_ = 59.291, *p* < 0.001). The treatment factor didn’t attain a significant level (*F*_(1,249)_ = 3.167, *p* = 0.081). The two groups had no difference in the SC per approach.

### Forced Swim Test

**Figure 6:**
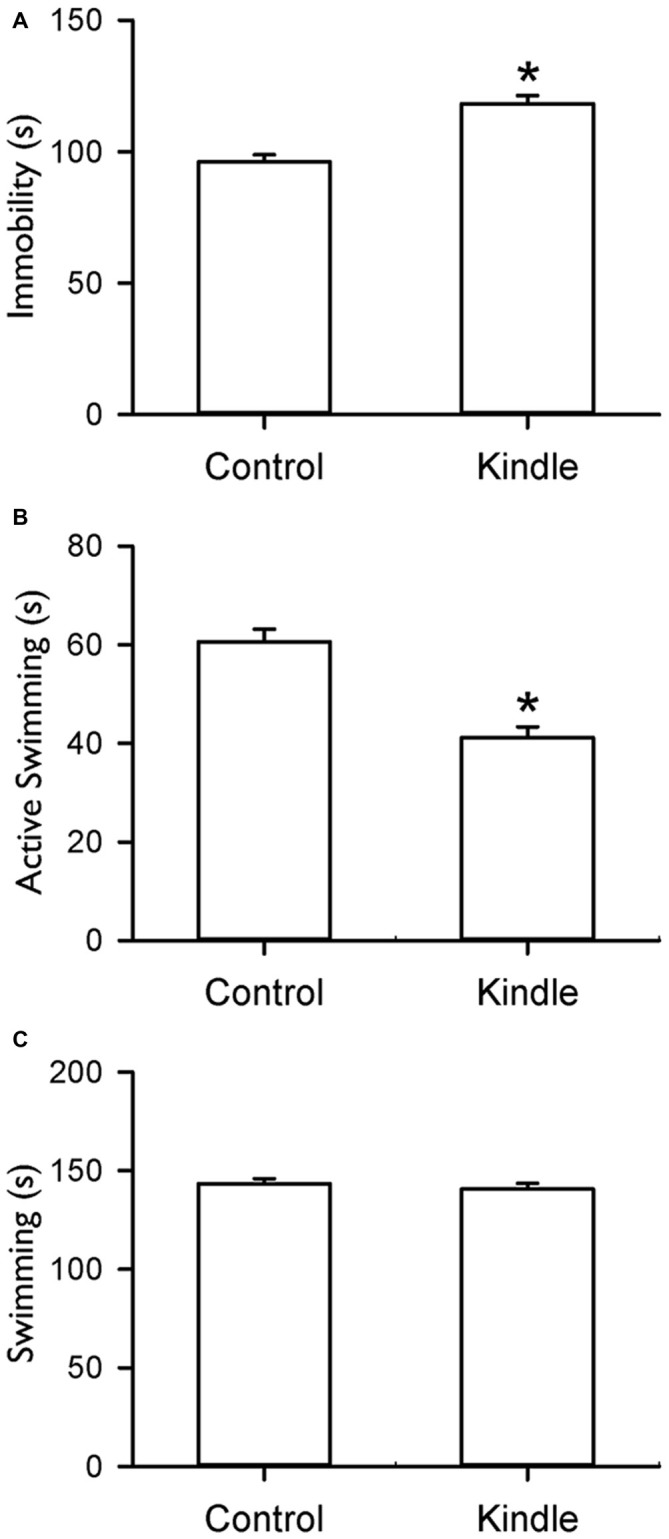
**Durations of immobility (A), active swimming (B) and swimming (C) of the FS test in the control and kindle groups.** The data are presented with the Mean ± SEM. *p < 0.05 vs. control.

Figure 6 shows indexes during the FS test. The kindle group showed a significantly longer immobility behavior compared to that of the control group (*t* = 5.334, *p* < 0.001). Twenty rats (80%) of the kindle group had longer immobility compared to the mean + 99% confidence interval of the control group (Table 1). The kindle group had a significantly shorter duration of active swimming than the control group (*t* = 5.72, *p* < 0.001). The duration of the swimming was not different between the two groups (*t* = 0.703, *p* = 0.486).

### Elevated Plus Maze Test

**Figure 7:**
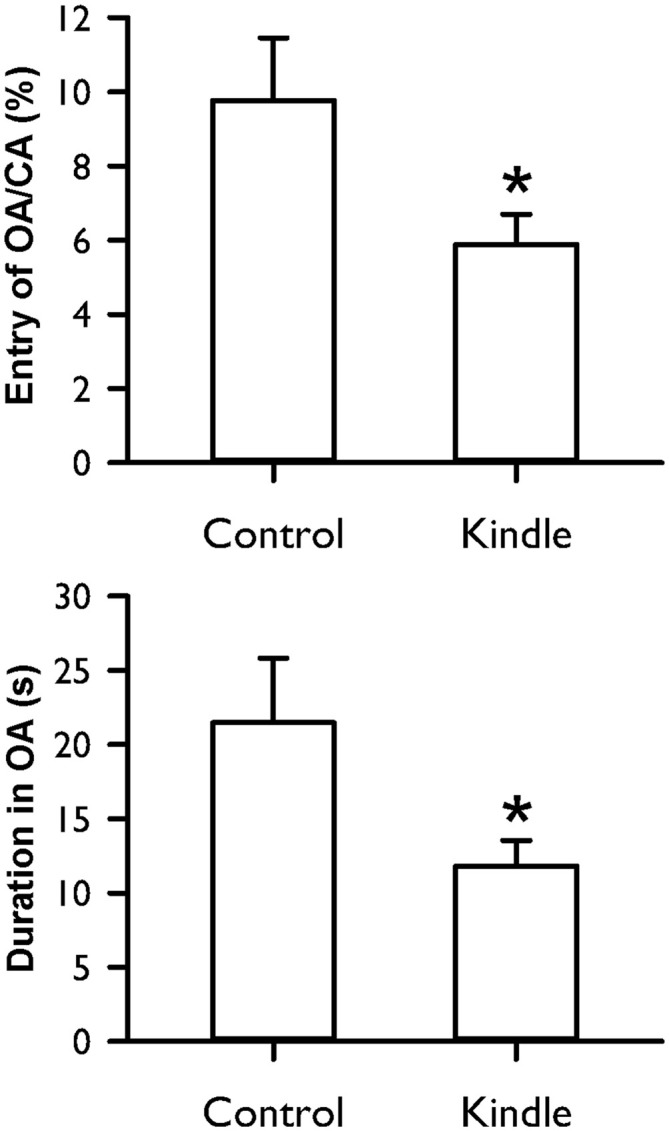
**Entry ratio of open arms (OAs)/closed arms (CAs) and duration of staying in OAs of the EPM test in the control and kindle groups.** The data are presented with the Mean ± SEM. *p < 0.05 vs. control.

Figure 7 shows the entry ratio of OAs/CAs and duration of staying OAs of an EPM. The kindle group had significantly shorter duration of staying in OAs than the control group (*t* = 2.068, *p* = 0.044). The entry ratio into OAs and CAs between the two groups was significantly different (*t* = 2.062, *p* = 0.044). More than 40% of the kindle group had lower value than the mean −99% confidence interval of the control group (Table 1). Total movement in the EPM was not different between the two groups (control, 19.8 ± 6.0 m; kindle, 19.5 ± 5.3 m; *t* = 0.137, *p* = 0.89).

### Open Field Test

**Figure 8:**
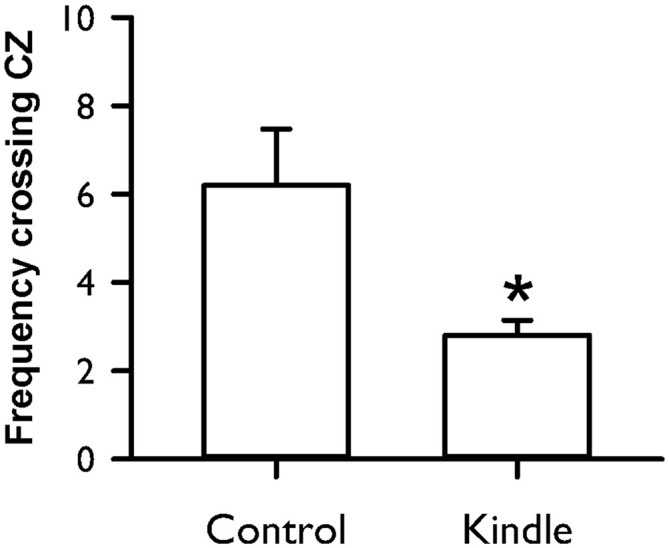
**Frequency crossing the central zone (CZ) of an OF in the control and kindle groups.** The data are presented with the Mean ± SEM. *p < 0.05 vs. control.

Figure 8 shows the frequency crossing the CZ of an OF cage. The kindle group had a significantly lower frequency crossing the CZ compared with the control group (*t* = 2.58, *p* = 0.015). Forty percent of the kindle group fell out the range of the mean −99% confidence interval of the control group (Table 1). Total movement in the OF apparatus showed no difference between the two groups (control, 29.5 ± 2.4 m; kindle, 29.1 ± 2.1 m; *t* = 0.137, *p* = 0.89).

## Discussion

The rapid amygdala kindling successully elicited ADs and afterwards caused a tonic-clonic motor seizure. The kindle group showed anxiety-like behavior in terms of low frequency entering open area of the EPM test and low frequency crossing CZ of the OF test. Moreover, the kindle group displayed low sucrose intake (anhedonia) in the SC test and long immobility (despair) in the FS test, which are indexes of depressive behaviors. Our results indicate that the rapid kindling of right-side amygdala aggravates anxiety- and depression-like behaviors. The rapid amygdala kindling model has a face validity as TLE patients.

Rats after the rapid amygdala kindling had the propensity to develop anxiety- and depression-like behaviors in terms of four behavioral tests. A considerable portion of patients with epilepsy experience cormobid anxiety (25–52%) or depression (16–50%) (Piazzini et al., 2001; Kanner, 2007; Desai et al., 2010; de Oliveira et al., 2010). In the current study, anxiety-like behavior occurred in 40–48% of the kindle group. Andedonia behavior showed in 40–48% of the kindle group, and despair-like behavior existed in 80% of the kindle group. There are similar prevalences to psychiatric comorbidity in patients with epilepsy and TLE-like model with rapid amygdala kindling. During the measurement period in the EPM or the OF, the total movement was not different in the two groups. Moreover, there was no change of normal swimming in the FS test, and exploring behavior (in terms of approach frequency) had no motor impairment in the SC test. These results suggest that the anxiety- and depression-like behaviors are related to the amygdala kindling.

Rapid amygdala kindling has the advantage that Racine’s stage 5 can occur within the first few days because up to 24 stimulations can be given each day (Lothman et al., 1985; Lothman and Williamson, 1994). Increased number of kindling stimulations were accompanied by long AD duration of both the amygdala and frontal cortex, which indicates a neural plasticity taking place in the amygdala and associated network, such as fiber sprouting (Ebert and Löscher, 1995; Smith et al., 2005). The phenomenon of increased fiber sprouting (Kandratavicius et al., 2012) or abnormal functional connectivity in the amygdalofrontal network (Doucet et al., 2013) is also observed in TLE patients. The rapid kindling process has been used to assess anticonvulsants or alternative therapeutics (De Smedt et al., 2007; Shahpari et al., 2012; Shojaei et al., 2014). The present study showed psychiatric comorbidity in rats with the rapid amygdala kindling. The data provides additional validation of the rapid amygdala kindling as a TLE-like model.

In a previous study (Jones et al., 2009), early maternal separation of rats at birth accelerated the kindling process. The rapid amygdala kindling resulted in an anxiolytic effect as evidenced by an increase in time spent in the OAs of the EPM compared with the sham group. In contrast, the present study showed AD with severe motor seizure and anxiety-like behavior after the rapid amygdala kindling of normal rats with no stress of early separation. Stress of early isolation or maternal separation often causes abnormal development of the limbic region (Kumar et al., 2011). The irregular limbic modulation may alter the level of psychomotor behavior. It may be a reason to explain the different effects of the rapid kindling on rats.

The present study used a re-test stimulation 60 min before the EPM test or the OF test to ascertain seizure severity in the kindle group exclusively. Animals with a Racine’s stage 5 motor seizure in response to the re-test stimulation were further analyzed. Because movement in either the EPM test or the OF test had no significant difference between the two groups, kindled animals thus have a low interest to enter OAs in the EPM test or to cross the CZ in the OF test. Before the anxiety behavioral tests, the re-test stimulation perhaps elicits a stressful event which may result in worsening the anxiety-like behavior. A further study needs to be done to determine the contribution of the re-test stimulation on anxiety-like behavior.

In our previous study, spike patterns of epileptiform discharges with regard to the severity of motor seizures have a similar development between conventional and rapid amygdala kindling (Wang et al., 2016). The development of AD and motor seizure stage seems to be similar in conventional kindling (Adamec and Morgan, 1994; Helfer et al., 1996) and rapid kindling (Jones et al., 2009) of the amygdala. An anxiogenic response is reported in the conventional amygdala kindling model (Adamec and Morgan, 1994; Helfer et al., 1996; Hannesson et al., 2008) or the pilocarpine- and kainic acid-induced TLE models (Inostroza et al., 2012). The effect of conventional amgdala kindling on anxiety-like behavior has been demonstrated to be related to stimulation side/site, gender, etc., (Adamec and Morgan, 1994; Kalynchuk, 2000; Wintink et al., 2003; Adamec et al., 2005). Furthermore, increases in emotionality or defensiveness are also accompanied by amygdala kindlings (Helfer et al., 1996). The reduction of exploring OAs of the EPM test in the amygdala kindling group is reversed by a benzodiazepam anxiolytic (Helfer et al., 1996). The amygdala is known as a fear and emotional circuit (Goddard, 1983). Accordingly, extensive stimulation of the amygdala causes dysregulations of the emotional network in convulsive animals.

Patients with TLE are often comorbid with major depression (Kanner, 2007; Kandratavicius et al., 2012). The intake of sucrose fluid within 15 min in the 5-day SC test, which is validated as a hedonic measure (Jones et al., 2008; Huang et al., 2012), was significantly lower in the kindle group compared to the control group. Loss of taste preference and low consumption of saccharin solution is reported in immature rats under rapid amygdala kindling (Mazarati et al., 2007). The results support that the rapid kindling model should be associated with TLE patients’ behaviors. However, an anhedonic phenomenon is debated in the pilocarpine- and kainic acid-induced TLE models (Mazarati et al., 2008; Inostroza et al., 2012; Tchekalarova et al., 2013; Klein et al., 2015). In contrast, conventional amygdala kindling had no significant difference in sucrose intake during the sucrose preference test (Helfer et al., 1996; Wintink et al., 2003; Adamec et al., 2004). Studies with insignificant findings only performed a 1-day measure of the sucrose intake. In this study, there was no significant difference at the first day in all indexes but revealed significance in the following days in the SC test (Figure 5). The pattern of changes of significance throughout the 5-day measures was also observed in the lamortrigine experiment (Huang et al., 2012). These results perhaps explain the controversial findings in the 1-day sucrose measure during previous studies.

The kindle group showed significantly longer immobility of the FS test compared to the control group. Immobility of the FS test is also related to seizure severity under a rapid kindling process (Mazarati et al., 2007). In a previous study (Helfer et al., 1996), immobility duration of the FS test in 10 right-amygdala kindled rats is higher but not significant than those of 10 non-kindled rats. A small sample size may cause low statistical power. No significant difference was reported in the immobility of the 10-min FS test whereas rats received kindling stimulations of the left amygdala (Wintink et al., 2003). The immobility of a 5-min FS test has been validated as an index of the despaired mood (Cryan et al., 2005; Huang et al., 2012). Kindled stimulations of the left-side and right-side amygdala produce different effects on anxiety-like behaviors (Adamec and Morgan, 1994; Adamec et al., 2005). Therefore, different measure duration of the FS test and stimulation site may account for the discrepancy between the present and previous studies. Normal swimming and active swimming in the FS test are increased by serotonergic and noradrenergic antidepressants, respectively (Cryan et al., 2005). The kindle group had an exclusive change in active swimming of the FS test, not in normal swimming. Our data may indicate that dysfunctions of noradrenergic neurons occur under rapid amygdala kindling.

A great portion (80%) of the kindle group had longer immobility than the control group. The sensitivity of the FS test seemed to be 2-fold higher than the anhedonic portion (~40%) using the SC test. The distribution of the portion with kindling-induced depression-like behaviors in the two behavioral tests remarkably differs from the approximate portion of the same two behavioral tests in an acid-induced muscle pain model (Liu et al., 2014). The immobility in the FS test can reflect an actively coping strategy to an inescapable situation (Cryan et al., 2005). The immobility during the FS test is related to not only “behavioral despair” but also “learned helplessness”. The “learned helplessness” usually leads to a loss of interest in work, life, etc. Afterward, individuals may evolve a feeling of hopelessness and reveal as immobility with sinking into water in the FS test with shorter duration of active swimmming in the kindle group (Figure 6). The results may partially support a several fold higher risk of suicide in patients with epilepsy (Kanner, 2007).

The present study used adult rats of 8–15 weeks old. The functional brain changes or neurodegenerative progression is related with age. The amygdala kindling threshold of adult rats is higher than that of immature animals (Moshe et al., 1981). The kindling-induced motor behaviors in adult rats are more reliable than those of neonate or infant rats (Gilbert and Cain, 1981; Yoshioka et al., 2000). Although the present study used animals with a large variance in age, we showed consistent kindling phenomenon and also observed severe anxiety-like or depressive-like comorbidity in a considerable portion of rats. So as to strengthen the effect of kindling on psychiatric comorbidity further, animals with a small age range may be helpful to elevate statistical power.

Several aspects of consistencies are found between the rapid kindling and conventional kindling, including AD progression (Wang et al., 2016) and anxiogenic effect (Adamec and Morgan, 1994; Helfer et al., 1996). However, there were subtle differences, for example, fiber sprouting (Ebert and Löscher, 1995). Different observations were also found between the rapid kindling and conventional kindling in the measure of depression-like behavior, such as sucrose intake (Helfer et al., 1996; Wintink et al., 2003; Adamec et al., 2004) and immobility during the FS test (Helfer et al., 1996). As we mentioned above, those studies have different measure parameters or small sample numbers, which may cause varied results. To further clarify the effect of amygdala kindling on depressive comorbidity, it will be important to assess same depresion-like behavior tests in animals with rapid kindling or conventional kindling in the future.

In summary, rapid amygdala kindling elicited a tonic-clonic stage 5 convulsive seizure. The current study provides further evidence on the propensity of anxiety- and depression-like behaviors in a considerable portion of the TLE-like model with rapid amygdala kindling.

## Author Contributions

S-DC: experimental design and writing of the article. Y-LW and S-FL: data analysis. F-ZS: experimental design, data analysis, and article draft.

## Conflict of Interest Statement

The authors declare that the research was conducted in the absence of any commercial or financial relationships that could be construed as a potential conflict of interest.
